# 

**Published:** 1877-10

**Authors:** 


ESTABLISHED IN 1855.

VOLUME XV | OCTOBER—1877 | NUMBER 7.

### ATLANTA

### Medical and Surgical Journal.

MONTHLY: THREE DOLLARS PER ANNUM, IN ADVANCE.

EDITOR:

J. G. WESTMORELAND, M.D.

*Professor Materia Medica in Atlanta Medical College.*

ASSOCIATE EDITORS:

R. W. WESTMORELAND, M.D.,

SHIRLEY BRAGG, M.D.,

H. H. DICKSON, PUBLISHER.

*PAX ET SCIENTIA, SED VERITAS SINE TIMORE.*

ATLANTA, GEORGIA:

*H. H. DICKSON, BOOK AND JOB PRINTER AND BOOKBINDER.*

1877.

